# Oglethorpe Medical and Surgical Journal

**Published:** 1858-05

**Authors:** 


### Oglethorpe Med. & Surg. Journal.

We have received the first number of the Oglethorpe Med. and Surg'l Journal, edited by H. L. Byrd, M. D., Prof. of the Principles and Practice of Medicine, in Oglethorpe Medical College, and Holmes Steele, M. D., Professor of Obstetrics and Diseases of women and children, in Oglethorpe Medical College.

This is a bi-monthly Journal of sixty-four pages, and is furnished to subscribers at $2 per annum, in advance.

"All communications intended for publication should be addressed to H. L. Byrd, M. D., Savannah, Ga., to whom, also, all moneys for subscription may be sent."

As we learn from the Journal, the issue at this time is the redemption of a promise made to the public about a year ago.

The Journal is highly creditable in its appearance, and contains a large amount of valuable and interesting matter.

We place it with pleasure upon our exchange list.

